# Chemical Composition and Antimicrobial Activity of Essential Oils from Three Mediterranean Plants against Eighteen Pathogenic Bacteria and Fungi

**DOI:** 10.3390/pharmaceutics14081608

**Published:** 2022-08-01

**Authors:** Soukaina Razzouk, Mouaad Amine Mazri, Lamya Jeldi, Bacem Mnasri, Lahcen Ouahmane, Mohamed Najib Alfeddy

**Affiliations:** 1 Plant Protection Research Unit, Regional Center of Agricultural Research of Marrakech, National Institute of Agricultural Research, Avenue Ennasr, BP 415 Rabat Principale, Rabat 10090, Morocco; razzouk.soukaina89@gmail.com (S.R.); lamyajeldi@gmail.com (L.J.); 2 Laboratory of Microbial Biotechnology, Agro-Sciences and Environment (BioMAgE), Cadi Ayyad University, Marrakesh 40000, Morocco; l.ouahmane@gmail.com; 3 Agro-Biotechnology Research Unit, Regional Center of Agricultural Research of Marrakech, National Institute of Agricultural Research, Avenue Ennasr, BP 415 Rabat Principale, Rabat 10090, Morocco; mouaadamine.mazri@inra.ma; 4 Center of Biotechnology of Borj-Cédria, Hammam-Lif 2050, Tunisia; mnbacemm@yahoo.com

**Keywords:** antibacterial activity, antifungal activity, chemical composition, essential oil, *Laurus nobilis*, *Syzygium aromaticum*, *Thymus leptobotrys*

## Abstract

The chemical composition and antimicrobial activity of essential oils (EOs) obtained from three medicinal plants of the Moroccan flora were evaluated. The chemical composition of EOs of *Thymus leptobotrys*, *Laurus nobilis* and *Syzygium aromaticum* was determined using a gas chromatograph coupled with mass spectrometry. Carvacrol (75.05%) was the main constituent of *T. leptobotrys* EOs, while 1,8-cineole (31.48%) and eugenol (82.16%) were the predominant components of *L. nobilis* and *S. aromaticum* EOs, respectively. The antimicrobial activity of the EOs was evaluated qualitatively and quantitatively against 18 microbial strains pathogenic to humans by using the disc diffusion method, and by measuring the minimum inhibitory concentration (MIC) and minimum microbicidal concentration (MMC). The EOs of *T. leptobotrys* were the most active against the strains tested, with inhibitory zone values ranging from 7.00 to 45.00 mm, and MIC and MMC values ranging from 0.312 to 80.00 mg/mL. In many cases, these EOs exhibited higher antibacterial and antifungal activities than the chemical compounds ciprofloxacin and fluconazole, respectively. This high antimicrobial activity can be ascribed to their richness in carvacrol. The EOs of *T. leptobotrys*, *L. nobilis*, and *S. aromaticum* could be considered a promising alternative to replace chemical antimicrobials, and a readily available natural source of bioactive compounds.

## 1. Introduction

Medicinal and aromatic plants are an interesting and abundant source of natural molecules of high relevance for different applications. The molecules derived from these plants possess specific properties and biological activities that determine the appropriateness of their use [1,2]. Medicinal plants have been used since ancient times in folk medicine and are used today to extract active ingredients with health-promoting properties [3,4].

In Morocco, there are more than 600 species used for medicinal purposes [5,6]. Among all these species, *Laurus nobilis*, *Thymus leptobotrys* and *Syzygium aromaticum* have been used for years in folk medicine due to their potent biological activities [7,8,9]. *Laurus nobilis* belongs to the Lauraceae family. It is an evergreen tree that can reach 12 m in height. In Morocco, it grows spontaneously in the forests of Eastern and Western Rif and the Middle Atlas. Its leaves are frequently used in folk medicine to treat gastrointestinal disorders and as a fragrance ingredient in the cosmetic and food industries [10]. The leaves of *L. nobilis* are also used in modern pharmaceutical industry [11]. Previous studies on the essential oils (EOs) from *L. nobilis* leaves have demonstrated their antimicrobial activity against a variety of pathogenic microorganisms, such as *Enterococcus faecalis*, *Salmonella pullorum, Listeria monocytogenes*, *Candida albicans*, *C. glabrata*, *C. parapsilosis*, *C. krusei*, *Micrococcus luteus*, *Bacillus subtilis*, *Klebsiella pneumoniae* and *Staphylococcus aureus* [10,12,13]. The chemical composition of *L. nobilis* EOs from different geographical origins has been studied. In all cases, 1,8-cineole was found to be the predominant component with percentages (34.6–56.0%) varying depending on the geographical origin of the plant [10,11,14,15,16].

*Thymus* is one of the most important genera of the family Lamiaceae. It is cultivated and naturally growing in many countries but mainly found in the Mediterranean region [17]. *Thymus* has been widely used in folk medicine due to the various therapeutic properties of its extracts, for example, antirheumatic, antiseptic, antispasmodic and antimicrobial properties [18]. The oil of *Thymus* is also used to strengthen the immune system [18]. *T. leptobotrys* is a *Thymus* species endemic to Morocco. It can reach 25 cm in height. It has elliptical green leaves. It is a species of arid and semi-arid climate zones that generally grows on rocky pastures, as well as calcareous or siliceous soils. This plant has been used for decades in different forms (powder, decoction or infusion) to treat various diseases, including digestive disorders, fever, cough and colds [19]. Previous researchers have shown that *T. leptobotrys* EOs have antifungal activities against *C. albicans*, *C. krusei*, *C. glabrata* and *C. parapsilosis* [20], and hamper the germination of *Penicillium digitatum*, *P. italicum* and *Geotrichum citri-aurantii* spores [21]. The EOs of *T. leptobotrys* also exhibited excellent antibacterial activity against *S. aureus*, *M. luteus*, *Bacillus cereus*, *Escherichia coli*, *Pseudomonas aeruginosa*, *K. pneumonia*, *Staphylococcus epidermidis*, *Acinetobacter baumaniin*, *Enterobacter cloacae*, *Citrobacter freundii*, *Salmonella* sp., and *Proteus mirabilis* [22,23]. The potent antifungal activity of Moroccan thyme EOs can be ascribed to their high content in carvacrol [20,21,22]. Indeed, analysis of the chemical composition of *T. leptobotrys* EOs from different geographic locations showed that carvacrol (73.6–79.1%) was the most abundant component [20,21,23,24].

Regarding *S. aromaticum*, it is a species of the Myrtaceae family. It is native to East Indonesia but grows spontaneously in many countries, including those of the Mediterranean region. It is an arborescent plant that can reach 10 m in height. It has oval leaves of up to 11 cm long. The flowers are yellow and small. The fruits are red ellipsoid drupes. *S. aromaticum* was previously studied for its antimicrobial, antioxidant, anticancer, anti-inflammatory and antidiabetic properties [25]. The EOs of *S. aromaticum* showed significant antibacterial activity against many pathogens, such as *Serratia* sp., *Salmonella* sp., *Kluyvera* sp., *Klebsiella* sp. and *E. coli* [26]. The EOs of *S. aromaticum* also exhibited potent antifungal activity against some common fungal species, such as *Fusarium moniliforme NCIM 1100*, *Fusarium oxysporum MTCC 284*, *Aspergillus* sp., *Mucor* sp., *Trichophyton rubrum* and *Microsporum gypseum* [27]. Eugenol was found to be the major compound in most *S. aromaticum* oils, with a concentration varying depending on the climatic and environmental conditions of the growing region, as well as the plant part and extraction method used [10,25,28,29].

One of today’s most pressing health issues is the emergence of many forms of resistant microorganisms. This is caused by the overuse of antibiotics, which has led to a decrease in their effectiveness [30]. For example, *E. coli* (strain *O157:H7*) is responsible for significant health emergencies worldwide, causing 2100 hospitalizations and 20 deaths each year [31]. The multidrug-resistant *S. aureus* is responsible for life-threatening pneumonia, necrotizing fasciitis and toxins [32]. To date, more than 17 different *Candida* species have been identified as bloodstream infection pathogens [33]. These species affect more than 250,000 people each year, with *C. albicans* being responsible for the majority of candidemia cases [33,34].

Based on the above, the aim of this work was to analyze the chemical composition of EOs from *L. nobilis*, *T. leptobotrys* and *S. aromaticum* by gas chromatography/mass spectrophotometry (GC/MS), and to evaluate their antimicrobial activity against 18 bacterial and yeast strains belonging to different genera. This investigation is of considerable interest by highlighting the chemodiversity of these species with high medicinal potential. It would also help to identify the most appropriate usage of these medicinal plants.

## 2. Materials and Methods

### 2.1. Chemicals

Hexane, anhydrous sodium sulfate, dimethylsulfoxide (DMSO), trypticase soy, Mueller–Hinton and Sabouraud dextrose were purchased from Sigma-Aldrich (Steinheim, Germany). Fluconazole and ciprofloxacin were purchased from Hi Media (Mumbai, India). Sodium chloride (NaCl) was purchased from local markets.

### 2.2. Plant Material

The leaves of *T. leptobotrys* and *L. nobilis* were collected during the flowering stage (May 2019) from their natural habitat in the regions of Tafraout (29°42′47.8″ N 8°58′51.7″ W; semi-tropical and continental climate; annual temperature range, 2–31 °C; the soil is mostly alluvial, consisting of sands and silts) and Beni Mellal (32°18′13.0″ N 6°15′27.0″ W; Mediterranean climate; annual temperature range, 3–40 °C; the soil is composed of limestone, marls, and sandstone), respectively. Fresh flower buds of *S. aromaticum* were purchased from an herbalist located in Marrakech, Morocco (31°37′44.3″ N 7°59′13.8″ W). The plant material was air-dried in a dark and humid environment before use.

### 2.3. Extraction and Characterization of EOs

Plant EOs were extracted using the hydrodistillation method in a Clevenger-type apparatus. Briefly, 200 g of the dried material of each species was subjected to three distillations of 3 h each. The EOs were extracted and dried with anhydrous sodium sulfate, then stored at 4 °C in the dark until use. Each extraction was performed in triplicate.

The yield of EOs was calculated using the following formula: RHE (%) = (V_HE_/DM) × 100, where RHE is the EO yield expressed as ml per 100 g dry matter; V_HE_ is the volume of EO recovered (mL) and DM is the amount of dry plant material used for extraction (g).

Chromatographic analyses were performed on a gas chromatograph (Perkin ElmerTM GC-680) coupled with mass spectrometry (Q-8 MS with ion trap). The fragmentation was carried out by electronic impact under a field of 70 eV. The capillary column used was an Agilent 19091S-433: 2169.66548 HP-5MS 5% Phenyl Methyl Silox (30 m × 250 µm). The film thickness was 0.25 µm. The column temperature was programmed from 50 to 325 °C at a rate of 4 °C/min. The carrier gas was helium whose flow rate was set at 1 mL/min. The sample injection worked in split mode. The device was connected to a computer system managing a NIST mass spectrum library. All chromatographic analyses were performed in triplicate.

### 2.4. Antimicrobial Activity of EOs

#### 2.4.1. Bacterial and Yeast Strains

The effects of EOs were evaluated on 13 bacterial strains and 5 yeasts: *Staphylococcus aureus* (209 PCIP 53156), *S. aureus* (ATCC 29213), *Micrococcus luteus* (ATCC381), *Bacillus cereus* (ATCC 14579), *Escherichia coli* (ATCC 8739), *E. coli (ATCC 35214)*, *Pseudomonas aeruginosa* (DSM 50090), *P. aeruginosa* (ATCC 27853), *Klebsiella pneumoniae* (CIP 104727), *K. pneumoniae* (clinical isolates), *Enterococcus faecalis* (ATCC 29212), *Listeria monocytogenes* (ATCC 19115), *Salmonella enteritidis* (DMB 560), *Candida albicans* (CCMM L4), *C. krusei* (CCMM L10), *C. glabrata* (CCMM L7), *C. parapsilosis* (CCMM L18) and *Aspergilus niger* (CCMM M100). The bacterial and yeast strains were provided by the Center of Biotechnology, Borj Cedria, Tunisia.

#### 2.4.2. Qualitative Analysis of Antimicrobial Activity: The Disc Diffusion Method

A sterile physiological water saline (9‰, NaCl) inoculum was made from a 24 h culture for bacteria and a 48 h culture for yeast. Afterwards, 0.1 mL of the inoculum was plated on Mueller–Hinton agar medium and Sabouraud dextrose agar medium for bacteria and yeasts, respectively. The plates were dried for 15 min and then sterile disks loaded with 2 µL EO and 10% DMSO were deposited on them. Additionally, two types of controls were used: a negative control with 2 µL DMSO, an antibiotic compound (ciprofloxacin, 15 µg/disc) for bacteria and an antifungal compound (fluconazole, 40 µg/disc) for yeasts. For bacteria, the plates were incubated at 37 °C for 24 h, while they were incubated at 25 °C for 48 h for yeasts. 

The diameter of the zone of inhibition was measured (mm) and classified as follows: Ø < 8 mm, non-sensitive; 8 ≤ Ø ≤ 14 mm, sensitive; 15 ≤ Ø ≤ 19 mm, very sensitive; and Ø ≥ 20 mm, extremely sensitive [35].

#### 2.4.3. Quantitative Analysis of Antimicrobial Activity

1. Determination of the minimum inhibitory concentration (MIC)

Using EOs and liquid trypticase soy medium supplemented with 2% DMSO, a series of mixtures of the two stock solutions was prepared to obtain a concentration range of EOs between 80 mg/mL and 0.312 mg/mL. From a 24 h old bacterial culture and 48 h old yeast culture, a microbial suspension was prepared in liquid trypticase soy medium. The suspension was adjusted to an optical density of 0.3 to reach the required concentration of 10^6^ Colony Forming Units (CFU)/mL for bacteria and 1–2 × 10^3^ cells/mL for fungi [36]. Afterwards, serial dilutions of EOs were prepared in sterile test tubes containing liquid Mueller–Hinton and Sabouraud dextrose media for bacteria and yeasts, respectively. Dilutions in sterile distilled water were made for ciprofloxacin and fluconazole. Micro-dilution plates containing 96 wells were prepared by dispensing 100 µL of the microbial suspension and 100 µL of each dilution of the EO or antibiotic into each well. The 96-well plates were then incubated at 37 °C for 24 h for bacteria, and at 25 °C for 48 h for fungi. The MIC was determined by selecting the lowest concentration of EO or antibiotic that inhibited microbial growth (absence of cloudiness).

2. Determination of the minimum microbicidal concentration (MMC)

Wells with no visible microbial growth were streaked onto Mueller–Hinton agar medium for bacteria and Sabouraud dextrose agar medium for yeasts. The MMC was defined as the lowest concentration of tested samples, showing no visible microbial growth after incubation at 37 °C for 24 h for bacteria, and at 25 °C for 48 h for yeasts.

### 2.5. Data Collection and Statistical Analysis

All experiments were made in triplicate. Data were reported as mean ± standard deviation. The significant difference among samples was determined by univariate analysis of variance followed by Tukey’s post-hoc test at 5% significance level using SPSS statistical software version 23 for Windows (IBM SPSS Inc., Chicago, IL, USA).

## 3. Results and Discussion

### 3.1. EO Yield and Chemical Composition

The results of this work showed that the yield of EOs varies depending on the species (Table 1). The highest value was observed in *S. aromaticum* (13.24%). The yield of EO of *L. nobilis* was 1.81%, while that of *T. leptobotrys* was 1.79%.

The yield and composition of EOs are multifactor dependent since they vary depending on the geographical origin of plants, climate and culture conditions, phenological stage, extraction method and among genotypes within the same species. Along this line, the yield of EOs from *S. aromaticum* (13.24%) and *L. nobilis* (1.81%) used in the present study were higher than those previously reported in the literature from other regions or obtained by using different extraction methods (10.54–11.6% and 0.57–0.95%, respectively) [8,10,26,37,38,39]. Regarding *T. leptobotrys* from Tafraout, the yield of EOs was 1.79%, lower than that (2.5%) observed by Oubihi et al. [23]. 

*L. nobilis* EOs contained 17 compounds. The main compound was 1,8-cineole (31.48%). This is in good agreement with the majority of studies found in the literature. However, it worth noting that the content of 1,8-cineole varies depending on the geographical location of the plant and its growing conditions. For example, the content of 1,8-cineole was found to be 56% in plants from Grombalia, Tunisia [11], 34.62% in those from Tizi-Ouzou, Algeria [15], while it ranged from 30.52% to 40.85% in *L. nobilis* plants grown in other regions of Morocco [12,40]. 1,8-Cineole is a saturated monoterpene found in many plant species [41]. It has been widely used in the pharmaceutical and cosmetic industries due to its numerous health-promoting properties, such as anti-ochratoxigenic, anti-inflammatory, antioxidant and antimicrobial activities [39,41]. Interestingly, 1,8-cineole was not found in the EOs of *T. leptobotrys* and *S. aromaticum* (Table 1). Indeed, the predominant compound in *T. leptobotrys* EOs was carvacrol (75.05%). A close value (79.1%) was obtained by Jamali et al. [36]. Carvacrol is a bioactive compound with a wide variety of biological properties including antioxidant, anti-inflammatory, anticarcinogenic, antiproliferative, antiplatelet and antimicrobial activities [20,23]. It is a major compound of many plant EOs, particularly those of the Labiatae family [42]. Due to its distinctive flavor and potent capacity to inhibit microorganism growth, carvacrol has been widely used as a food flavoring ingredient and preservative [43]. The main compound found in *S. aromaticum* EOs was eugenol (82.16%). This value is higher than that (61.42%) observed by Lambert et al. [29] in *S. aromaticum* from Sao Paulo (Brazil). Eugenol was not detected in the EOs of *T. leptobotrys*, while it was present in a low concentration (5.05%) in the EOs of *L. nobilis*. Eugenol is a functional component in numerous products [44]. It has been used for antibacterial, anti-inflammatory, analgesic, antioxidant, anticancer and antiseptic purposes [45,46]. Furthermore, eugenol is frequently used in agro-industrial applications to protect foods during storage from pathogens, and as a pesticide and fumigant [45]. The presence of monoterpenol, phenol and phenylpropene families in the EOs of the evaluated species is a good indicator of their potent antimicrobial activity [47,48,49].

In sum, our findings along with those from the literature provide evidence that the EO yield and composition of a given species vary depending on genetic and environmental factors. These differences would certainly affect the biological activity of EOs. Additionally, our results highlight the chemodiversity of these plants with high medicinal values. This would help to identify the most appropriate use of each species.

### 3.2. Antifungal Activity of EOs

#### 3.2.1. The Disk Diffusion Method

**Figure 1:**
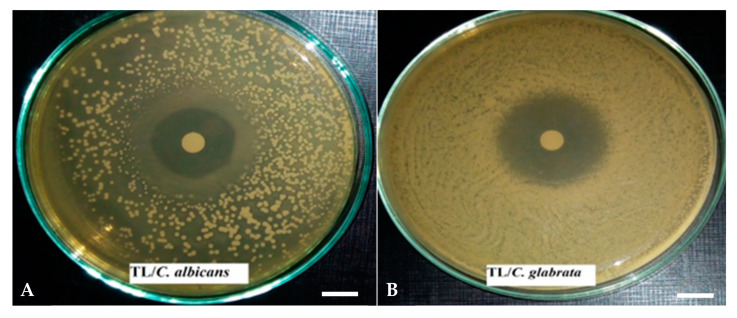
Examples of the antimicrobial activity of plant essential oils by the disc diffusion method. (**A**) Effect of *Thymus leptobotrys* on *Candida albicans*. (**B**) Effect of *Thymus leptobotrys* on *Candida glabrata*. Bars correspond to 1 cm.

The antifungal activity of EOs was evaluated qualitatively by the presence or absence of inhibition zones, and quantitatively by determining the MIC and MMC values. The findings of the disc diffusion method showed that all EOs evaluated had an inhibitory action against the growth of yeasts, with inhibition zone diameters ranging from 7.00 to 45.00 mm (Table 2). In many cases, this activity was more potent than that of fluconazole. The EOs of *T. leptobotrys* showed the strongest antifungal activity (Figure 1) against all the tested fungi (23.67–45.00 mm diameter of inhibition), whereas those of *L. nobilis* exhibited the weakest inhibitory effects (7.00–12.00 mm diameter of inhibition). Additionally, *A. niger* showed the highest sensitivity to EOs (12.00–45.00 mm diameter of inhibition) compared to *Candida* yeasts. Our findings are not consistent with those of Taarabt et al. [40] who observed a high susceptibility of *C. albicans* to *L. nobilis* EOs (10 µL/disc). This can be explained by different factors, such as the composition of EOs, the geographical location of plants and its environmental conditions, extraction method and the concentration of EO used [50].

#### 3.2.2. Determination of MIC and MMC Values

The MIC and MMC values varied depending on the strain and plant species (Table 3). The MIC values of *S. aromaticum* EOs ranged from 1.25 to 5.00 mg/mL. The MIC values of *T. leptobotrys* EOs varied from 0.625 to 1.25 mg/mL while those of *L. nobilis* exhibited a MIC range of 10.00–40.00 mg/mL. All MMC values were similar to MIC values, except for *C. parapsilosis* (Table 3). This highlights the high fungicidal activity of the EOs tested. Here again, the EO of *T. leptobotrys* were the most active extracts against all fungal strains, which confirms the results obtained by the disc diffusion method. This may be due to its high content in carvacrol (75.05%), which was absent in the other oils. This is in good agreement with the findings of Jamali et al. [20,22,36] and Boubaker et al. [21]. Carvacrol was found to exhibit a potent antifungal activity against other fungi and *Candida* species such as *C. tropicalis*, *C. lusitaniae*, *C. famata*, *Saccharomyces cerevisiae*, *P. digitatum*, *P. italicum* and *G. citri-aurantii* [7,21,23]. According to Di Pasqua et al. [51], carvacrol interacts with the cytoplasmic membrane through the acyl chains of phospholipids. This may cause the disruption of the permeability and fluidity of the cytoplasmic membrane.

### 3.3. Antibacterial Activity of EOs

#### 3.3.1. The Disk Diffusion Method

The results of the disc diffusion method are shown in Table 4. Almost all the EOs exhibited inhibitory activities against the growth of bacterial strains, with inhibition diameters ranging from 7.00 to 36.67 mm. Indeed, only *L. nobilis* EOs did not show an inhibitory effect against *P. aeruginosa* strains. On the other hand, the EOs of *L. nobilis* showed strong inhibitory effects against *S. aureus* (209 PCIP 53156), with an inhibition zone diameter of 32.33 mm, which is close to the value (40.00 mm) reported by Riabov et al. [50]. Here again, *T. leptobotrys* EOs exhibited the most potent activity against the bacterial strains evaluated. In many cases, this activity was higher than that of ciprofloxacin. Moreover, *T. leptobotrys* EOs were able to inhibit the growth of *E. coli* (ATCC 8739), *K. pneumoniae* (CIP 104727) and *L. monocytogenes* (ATCC 19115), even though these bacteria showed resistance to ciprofloxacin (Table 4). This highlights the potent antimicrobial activity of *T. leptobotrys* EOs against different types of pathogens (i.e., fungi, Gram-negative and Gram-positive bacteria), and suggests the use of these biological extracts as an alternative to chemical antibiotics.

#### 3.3.2. Determination of MIC and MMC Values

The MIC and MMC values varied depending on the EO and bacterial strain tested (Table 5). The EOs of *T. leptobotrys* and *S. aromaticum* showed greater antibacterial effects than those of *L. nobilis*, with similar MIC and MMC values in most cases. This demonstrates the potent bactericidal activity of these EOs. Several works have reported the strong antibacterial activity of *T. leptobotrys* and *S. aromaticum* EOs [23,26]. This may be due to their richness in phenolic compounds, including eugenol and carvacrol, and its precursor p-cymene [23,25,36,52]. Carvacrol was reported to have a high antimicrobial activity [17]. However, some researchers reported that the biological activities of EOs are due to the synergy between the major compounds rather than a single one [8,53]. Along this line, Jamali et al. [36] reported that the antimicrobial activity is more likely due to the interaction between carvacrol and thymol rather than carvacrol alone.

It is well known that Gram-positive bacteria are more sensitive to plant EOs than Gram-negative bacteria [54]. This is due to their cell walls. Indeed, Gram-positive bacteria have a cell wall that facilitates the action of EOs due to a strong incorporation of their active molecules into the wall surface [55]. The EO constituents cross cell membranes and cause an imbalance of potassium ions and intracellular ATP, which leads to microbial death [18,24,28,36,52]. In the present study, both Gram-positive and Gram-negative bacteria showed remarkable sensitivity to *T. leptobotrys* and *S. aromaticum* EOs, except for *P. aeruginosa* (DSM 50090) (Table 5). Interestingly, *P. aeruginosa* (ATCC 27853) was sensitive to *T. leptobotrys* and *S. aromaticum* EOs. This result highlights the fact that different bacterial strains of the same species may exhibit different levels of sensitivity/resistance to plant EOs [56,57]. Therefore, more studies should be carried out to identify potent natural compounds against *P. aeruginosa* (DSM 50090).

## 4. Conclusions

Nowadays, antimicrobial resistance is considered a major health concern that requires the development and use of novel therapeutic alternatives. Medicinal plants constitute a natural and abundant source of bioactive molecules with potent antimicrobial activity. The present study investigated the chemical composition and antimicrobial activity of three medicinal plants against several human pathogenic bacteria and fungi. It was found that the yield and composition of plant EOs, as well as their biological activities may considerably vary depending on the geographical origin of plants, climate and culture conditions, plant genotype and extraction method. The EOs obtained from *T. leptobotrys* were the most active against the strains tested. These EOs showed high levels of carvacrol, which was not detected in those obtained from *L. nobilis* and *S. aromaticum*. This suggests that carvacrol is most likely related to the antimicrobial activity of *T. leptobotrys*. The findings of this work open new perspectives for the potential use of these plants as cheap and effective agents against many human pathogens. Future research could focus on the domestication and production of secondary metabolites from these plants through tissue culture.

## Figures and Tables

**Table 1:** Yield and composition of essential oils.

Compound	RI ^1^	RT ^2^	Content (%)		
			*T. leptobotrys*	*L. nobilis*	*S. aromaticum*
α-Pinene	936	5.452	1.77	1.66	- ^3^
Sabinene	975	6.183	-	4.08	-
β-Pinene	980	6.266	-	1.45	-
Camphene	998	5.740	1.55	12.94	0.21
α-Terpinene	1020	7.005	0.76	-	-
ρ-Cymene	1025	7.160	5.71	-	-
γ-Terpinene	1059	7.849	5.79	-	-
1,8-Cineole	1069	7.323	-	31.48	-
Linalool	1099	8.659	-	12.13	-
Camphor	1168	9.665	2.10	-	0.29
4-Terpineol	1179	10.316	-	1.32	-
α-Terpineol	1196	10.571	-	7.85	-
Bornyl acetate	1279	12.467	-	1.85	-
Carvacrol	1302	12.696	75.05	-	-
Thymol	1316	12.483	2.46	-	-
Eugenol	1358	13.819	-	5.05	82.16
Trans-isoeugenol	1406	15.515	-	1.88	-
α-Gurjunene	1407	16.408	1.12	-	-
β-Elemene	1417	14.517	-	1.61	-
Caryophyllene	1426	15.072	1.82	-	0.79
Aromadendrene	1445	15.423	1.13	-	-
Trans-cinnamyl acetate	1448	15.398	-	1.37	-
Methyleugenol	1499	14.650	-	9.61	-
β-Bisabolene	1511	16.554	0.73	-	-
Acetyleugenol	1521	16.855	-	-	16.55
Trans-isoelemicin	1568	18.909	-	1.69	-
β-Eudesmol	1635	19.051	-	0.89	-
(rac)-1,3,6,8-Tetramethylcyclododeca-1,2,6,7-tetraene	1670	19.510	-	3.14	-
Yield (%, *v*/*w*)			1.79 ± 0.06	1.81 ± 0.03	13.24 ± 0.01

^1^ Retention index measured relative to n-alkanes (C-9 to C-24) on the non-polar DB-5 column. ^2^ Retention time. ^3^ Compound not detected.

**Table 2:** Diameter of inhibition zones (mm) of essential oils against fungal strains.

Microorganism	Diameter in mm ^1^				
	Essential Oils (2 µL/disc)			Positive Control (40 µg/disc)	Negative Control (2 µL/disc)
	*S. aromaticum*	*T. leptobotrys*	*L. nobilis*	Fluconazole	DMSO
*C. albicans* (CCMM L4)	14.00 ± 1.00 d	27.00 ± 1.00 g	7.00 ± 0.00 a	26.00 ± 1.00 f,g	NA ^2^
*C. glabrata* (CCMM L7)	24.67 ± 0.58 f	30.67 ± 1.15 h	10.33 ± 0.58 b	21.33 ± 0.58 e	NA
*C. krusei* (CCMM L10)	20.33 ± 0.58 e	30.00 ± 1.00 h	9.33 ± 0.58 b	19.33 ± 0.58 e	NA
*C. parapsilosis* (CCMM L18)	25.00 ± 0.00 f	23.67 ± 0.58 f	9.00 ± 0.00 b	18.33 ± 0.58 e	NA
*A. niger* (CCMM M100)	44.00 ± 1.00 i	45.00 ± 1.00 i	12.00 ± 0.00 c	6.67 ± 0.58 a	NA

Data are means ± standard deviations (*n* = 3). Values followed by the same letters are not significantly different (*p* > 0.05) by the Tukey’s post-hoc test. ^1^ Disc diameter included (6 mm). ^2^ NA: not active.

**Table 3:** Minimum inhibitory concentration (MIC) and minimum microbicidal concentration (MMC) of essential oils against fungal strains.

Microorganism		Essential Oils (mg/mL)			Positive Control (µg/mL)
	Antimicrobial Activity	*S. aromaticum*	*T. leptobotrys*	*L. nobilis*	Fluconazole
*C. albicans* (CCMM L4)	MIC	5.00 ± 0.00 c	1.25 ± 0.00 a	20.00 ± 0.00 e	0.24 ± 0.00
	MMC	5.00 ± 0.00 χ	1.25 ± 0.00 α	20.00 ± 0.00 δ	0.24 ± 0.00
*C. glabrata* (CCMM L7)	MIC	2.50 ± 0.00 b	1.25 ± 0.00 a	40.00 ± 0.00 f	0.24 ± 0.00
	MMC	2.50 ± 0.00 β	1.25 ± 0.00 α	40.00 ± 0.00 ε	0.24 ± 0.00
*C. krusei* (CCMM L10)	MIC	1.25 ± 0.00 a	1.25 ± 0.00 a	40.00 ± 0.00 f	0.24 ± 0.00
	MMC	1.25 ± 0.00 α	1.25 ± 0.00 α	40.00 ± 0.00 ε	0.24 ± 0.00
*C. parapsilosis* (CCMM L18)	MIC	1.25 ± 0.00 a	1.25 ± 0.00 a	10.00 ± 0.00 d	0.24 ± 0.00
	MMC	2.50 ± 0.00 β	1.25 ± 0.00 α	20.00 ± 0.00 δ	0.24 ± 0.00
*A. niger* (CCMM M100)	MIC	1.25 ± 0.00 a	0.625 ± 0.00 a	40.00 ± 0.00 f	NA ^1^
	MMC	1.25 ± 0.00 α	0.625 ± 0.00 α	40.00 ± 0.00 ε	NA

Data are means ± standard deviations (*n* = 3). Values followed by the same letters (for MIC values) or symbols (for MMC values) are not significantly different (*p* > 0.05) by the Tukey’s post-hoc test. ^1^ NA: not active.

**Table 4:** Diameter of inhibition zones (mm) of essential oils against bacterial strains.

Microorganism	Diameter in mm ^1^				
	Essential Oils (2 µL/disc)			Positive Control	Negative Control
	*S. aromaticum*	*T. leptobotrys*	*L. nobilis*	Ciprofloxacin (15 µg/disc)	DMSO (2 µL/disc)
Gram-negative bacteria					
*E. coli* (ATCC 35214)	12.67 ± 1.15 d,e	24.33 ± 3.05 h–j	8.33 ± 0.58 a–c	26.33 ± 2.30 j	NA ^2^
*E. coli* (ATCC 8739)	12.33 ± 0.58 c–e	20.33 ± 0.58 f,g	9.33 ± 0.58 a–d	6.00 ± 0.00 a	NA
*K. pneumoniae* (CIP 104727)	10.00 ± 1.00 a–d	23.67 ± 0.58 g–j	8.33 ± 0.58 a–c	6.00 ± 0.00 a	NA
*K. pneumoniae* (clinical isolates)	11.67 ± 0.58 b–d	22.33 ± 0.58 g–i	10.33 ± 0.58 a–d	10.00 ± 0.00 a–d	NA
*L. monocytogenes* (ATCC 19115)	15.00 ± 2.65 e	21.67 ± 4.93 g,h	8.00 ± 1.00 a,b	6.00 ± 0.00 a	NA
*S. enteritidis* (DMB 560)	11.33 ± 0.58 b–d	25.33 ± 1.15 i,j	9.67 ± 2.89 a–d	24.67 ± 3.05 h–j	NA
*P. aeruginosa* (ATCC 27853)	9.67 ± 0.58 a–d	20.67 ± 1.52 f,g	6.00 ± 0. 00 a	11.67 ± 0.58 b–d	NA
*P. aeruginosa* (DSM 50090)	6.33 ± 0.58 a	7.00 ± 0.00 a	6.00 ± 0.00 a	10.17 ± 0.21 a–d	NA
Gram-positive bacteria					
*S. aureus* (ATCC 29213)	11.67 ± 0.58 b–d	24.33 ± 1.15 h–j	7.67 ± 0.58 a,b	22.67 ± 0.58 g–i	NA
*S. aureus* (209 PCIP 53156)	21.67 ± 0.58 g,h	36.67 ± 0.58 l	32.33 ± 0.58 k	13.97 ± 0.06 d,e	NA
*B. cereus* (ATCC 14579)	9.67 ± 1.15 a–d	22.33 ± 2.08 g–i	8.00 ± 0.00 a,b	29.00 ± 1.00 k	NA
*M. luteus* (ATCC381)	26.33 ± 0.58 j	31.00 ± 1.73 k	10.33 ± 0.58 a–d	12.07 ± 0.12 c–e	NA
*E. faecalis* (ATCC 29212)	9.00 ± 1.00 a–d	18.33 ± 1.15 f	7.67 ± 1.15 a,b	21.00 ± 2.64 g	NA

Data are means ± standard deviations (*n* = 3). Values in the same column followed by the same letters are not significantly different (*p* > 0.05) by the Tukey’s post-hoc test. ^1^ Disc diameter included (6 mm). ^2^ NA: not active.

**Table 5:** Minimum inhibitory concentration (MIC) and minimum microbicidal concentration (MMC) against bacterial strains.

Microorganism		Essential Oils (mg/mL)			Positive Control (µg/mL)
	Antimicrobial Activity	*S. aromaticum*	*T. leptobotrys*	*L. nobilis*	Ciprofloxacin
Gram-negative bacteria					
*E. coli (ATCC 35214)*	MIC	1.25 ± 0.00 b	0.312 ± 0.00 a	10.00 ± 0.00 e	0.43 ± 0.00
	MMC	2.50 ± 0.00 χ	0.312 ± 0.00 α	20.00 ± 0.00 φ	0.43 ± 0.00
*E. coli (ATCC 8739)*	MIC	5.00 ± 0.00 d	2.50 ± 0.00 c	80.00 ± 0.00 h	0.85 ± 0.00
	MMC	5.00 ± 0.00 δ	2.50 ± 0.00 χ	80.00 ± 0.00 η	0.85 ± 0.00
*K. pneumoniae (CIP 104727)*	MIC	1.25 ± 0.00 b	0.625 ± 0.00 a	20.00 ± 0.00 f	0.85 ± 0.00
	MMC	2.50 ± 0.00 χ	0.625 ± 0.00 α	40.00 ± 0.00 γ	0.85 ± 0.00
*K. pneumoniae (clinical isolates)*	MIC	2.50 ± 0.00 c	2.50 ± 0.00 c	20.00 ± 0.00 f	225.00 ± 0.00
	MMC	2.50 ± 0.00 χ	2.50 ± 0.00 χ	20.00 ± 0.00 φ	225.00 ± 0.00
*L. monocytogenes (ATCC 19115)*	MIC	1.25 ± 0.00 b	0.312 ± 0.00 a	20.00 ± 0.00 f	14.06 ± 0.00
	MMC	2.50 ± 0.00 χ	0.312 ± 0.00 α	40.00 ± 0.00 γ	14.06 ± 0.00
*S. enteritidis (DMB 560)*	MIC	1.25 ± 0.00 b	0.625 ± 0.00 a	20.00 ± 0.00 f	14.06 ± 0.00
	MMC	2.50 ± 0.00 χ	0.625 ± 0.00 α	40.00 ± 0.00 γ	14.06 ± 0.00
*P. aeruginosa (ATCC 27853)*	MIC	1.25 ± 0.00 b	0.625 ± 0.00 a	20.00 ± 0.00 f	3.51 ± 0.00
	MMC	2.50 ± 0.00 χ	0.625 ± 0.00 α	20.00 ± 0.00 φ	3.51 ± 0.00
*P. aeruginosa (DSM 50090)*	MIC	80.00 ± 0.00 h	80.00 ± 0.00 h	80.00 ± 0.00 h	28.12 ± 0.00
	MMC	80.00 ± 0.00 η	80.00 ± 0.00 η	80.00 ± 0.00 η	28.12 ± 0.00
Gram-positive bacteria					
*S. aureus (ATCC 29213)*	MIC	1.25 ± 0.00 b	0.625 ± 0.00 a	40.00 ± 0.00 g	7.03 ± 0.00
	MMC	1.25 ± 0.00 β	0.625 ± 0.00 α	40.00 ± 0.00 γ	7.03 ± 0.00
*S. aureus (209 PCIP 53156)*	MIC	5.00 ± 0.00 d	1.25 ± 0.00 b	20.00 ± 0.00 f	225.00 ± 0.00
	MMC	5.00 ± 0.00 δ	1.25 ± 0.00 β	20.00 ± 0.00 φ	225.00 ± 0.00
*B. cereus (ATCC 14579)*	MIC	1.25 ± 0.00 b	0.312 ± 0.00 a	10.00 ± 0.00 e	0.85 ± 0.00
	MMC	2.50 ± 0.00 χ	0.312 ± 0.00 α	10.00 ± 0.00 ε	0.85 ± 0.00
*M. luteus (ATCC381)*	MIC	5.00 ± 0.00 d	2.50 ± 0.00 c	10.00 ± 0.00 e	225.00 ± 0.00
	MMC	5.00 ± 0.00 δ	2.50 ± 0.00 χ	10.00 ± 0.00 ε	225.00 ± 0.00
*E. faecalis (ATCC 29212)*	MIC	1.25 ± 0.00 b	0.625 ± 0.00 a	40.00 ± 0.00 g	0.43 ± 0.00
	MMC	2.50 ± 0.00 χ	0.625 ± 0.00 α	40.00 ± 0.00 γ	0.43 ± 0.00

Data are means ± standard deviations (*n* = 3). Values followed by the same letters (for MIC values) or symbols (for MMC values) are not significantly different (*p* > 0.05) by the Tukey’s post-hoc test.

## Data Availability

Data available on request.
